# Combination Treatment of Topical Imiquimod Plus Anti-PD-1 Antibody Exerts Significantly Potent Antitumor Effect

**DOI:** 10.3390/cancers13163948

**Published:** 2021-08-05

**Authors:** Kazumasa Oya, Yoshiyuki Nakamura, Zhu Zhenjie, Ryota Tanaka, Naoko Okiyama, Yuki Ichimura, Yosuke Ishitsuka, Akimasa Saito, Noriko Kubota, Rei Watanabe, Hideaki Tahara, Manabu Fujimoto, Yasuhiro Fujisawa

**Affiliations:** 1 Department of Dermatology, Faculty of Medicine, University of Tsukuba, Tsukuba 305-8575, Japan; oya_kazumasa@hotmail.com (K.O.); ketsu-@outlook.com (Z.Z.); ryota621@hotmail.co.jp (R.T.); naoko.okiyama@md.tsukuba.ac.jp (N.O.); k.himura.knzw@gmail.com (Y.I.); yosuke.ishitsuka@md.tsukuba.ac.jp (Y.I.); akisaitou-tuk@umin.ac.jp (A.S.); kubota.noriko@indigo.plala.or.jp (N.K.); rwatanabe@md.tsukuba.ac.jp (R.W.); fujimoto@derma.med.osaka-u.ac.jp (M.F.); fujisan@md.tsukuba.ac.jp (Y.F.); 2 Project Division of Cancer Biomolecular Therapy, Institute of Medical Science, The University of Tokyo, Tokyo 108-8639, Japan; tahara@ims.u-tokyo.ac.jp; 3 Department of Cancer Drug Discovery and Development, Osaka International Cancer Center, Osaka 541-8567, Japan

**Keywords:** imiquimod, anti-PD-1 antibody, combination therapy, melanoma

## Abstract

**Simple Summary:**

Imiquimod (IMQ), a synthetic ligand of Toll-like receptor 7, is known to exert antitumor effects. However, the exact mechanisms of the IMQ-induced antitumor effect have not been fully understood. Although both topical IMQ treatment and anti-PD-1 antibody may be used for primary skin lesions or skin metastases of various cancers, the efficacy of each monotherapy for these lesions is insufficient. In our study using a murine tumor model, we show that IFN-γ produced by CD8^+^ T cells may play a crucial role in the IMQ-induced antitumor effect. In addition, IMQ upregulated PD-1 expression in T cells as well as PD-L1/PD-L2 expression in myeloid cells. Furthermore, we also found that combination therapy of topical IMQ with anti-PD-1 antibody exerted a significantly potent antitumor effect when compared with each single therapy. Therefore, the combination therapy of topical IMQ plus anti-PD-1 antibody is promising therapy for skin cancer.

**Abstract:**

The exact mechanisms of the imiquimod (IMQ)-induced antitumor effect have not been fully understood. Although both topical IMQ treatment and anti-PD-1 antibody may be used for primary skin lesions or skin metastases of various cancers, the efficacy of each monotherapy for these lesions is insufficient. Using a murine tumor model and human samples, we aimed to elucidate the detailed mechanisms of the IMQ-induced antitumor effect and analyzed the antitumor effect of combination therapy of topical IMQ plus anti-PD-1 antibody. Topical IMQ significantly suppressed the tumor growth of MC38 in wildtype mice. IMQ upregulated interferon γ (IFN-γ) expression in CD8^+^ T cells in both the lymph nodes and the tumor, and the antitumor effect was abolished in both Rag1-deficient mice and IFN-γ-deficient mice, indicating that IFN-γ produced by CD8^+^ T cells play a crucial role in the IMQ-induced antitumor effect. IMQ also upregulated PD-1 expression in T cells as well as PD-L1/PD-L2 expression in myeloid cells, suggesting that IMQ induces not only T-cell activation but also T-cell exhaustion by enhanced PD-1 inhibitory signaling. Combination therapy of topical IMQ plus anti-PD-1 antibody exerted a significantly potent antitumor effect when compared with each single therapy, indicating that the combination therapy is a promising therapy for the skin lesions of various cancers.

## 1. Introduction

Toll-like receptor (TLR) signaling induces myeloid cell activation, connecting innate and adaptive immune responses, and TLR ligands have been proposed for use as agents in antitumor regimens for boosting adaptive immunity in cancer therapy [1]. TLR 7 and 8, which are mainly expressed in myeloid cells, play important roles in the activation of innate immunity by their recognition of single-stranded RNA [2]. Imiquimod (IMQ) is a synthetic ligand of TLR 7 that exerts antitumor and antiviral effects [3,4,5]. Several molecules such as type I interferon (IFN), IFN-γ, CCL2, IL-6, and tumor necrosis factor-related apoptosis-inducing ligand (TRAIL) are induced by TLR 7/8 agonists and have been speculated to have roles in the antitumor effect [6,7,8,9,10,11,12]. A previous report has also demonstrated that decreased production of CCL22 from tumor-associated macrophages by IMQ stimulation may play crucial roles in the antitumor effect [13]. However, the exact mechanisms of the IMQ-induced antitumor effect remain to be fully understood.

Topical 5% IMQ cream (Beselna Cream™) has already been approved by the US Food and Drug Administration for treatment of superficial basal cell carcinoma and actinic keratosis and has shown significant antitumor effects [3]. Topical IMQ has also been tried for use in the treatment of unresectable primary lesions or in-transit metastases (ITMs), skin metastases located predominantly in the superficial lymphatics between the primary and draining lymph nodes (LNs), of malignant melanoma [14,15]. Moreover, topical IMQ has been applied to skin metastases of other cancers such as breast cancer [16,17]. However, the efficacy of topical IMQ for these cutaneous lesions is insufficient [14,15,16,17].

PD-1, which is expressed mainly in T cells, plays crucial roles in the induction and maintenance of immune tolerance by binding to its ligands, PD-L1/PD-L2 [18]. Previous studies demonstrated that inhibition of the PD-1 pathway promotes effective immune responses against cancer cells [18], and PD-1 antibodies have demonstrated significant clinical efficacy in the treatment of several advanced cancers including malignant melanoma, lung cancer, and breast cancer [18]. Although clinical trials also demonstrated that anti-PD-1 antibodies improve the survival of advanced melanoma patients, the overall response rate (ORR) is around 40% and many lesions including ITM do not achieve an objective response [19,20]. The ORR of other cancers treated with anti-PD-1 antibodies is even lower than that of melanoma [21,22]. Since the cutaneous lesions of these malignancies frequently cause significant morbidity [15,23,24,25], a novel approach to the treatment of the lesions is necessary.

Because IMQ activates innate immunity, we speculated that combination therapy with topical IMQ may enhance the antitumor effect of anti-PD-1 antibody and that such combination therapy may exert a prominent antitumor effect. In this study, we aimed to elucidate the detailed mechanisms of the IMQ-induced antitumor effect and analyzed the antitumor effect of combination therapy of anti-PD-1 antibody and topical IMQ.

## 2. Materials and Methods

### 2.1. Mice

C57BL/6J mice raised under specific pathogen-free conditions were purchased from CLEA Japan (Tokyo, Japan). IFN-γ deficient mice, Rag1-deficient mice, and homozygous CD19-Cre transgenic mice were purchased from the Jackson Laboratory (Bar Harbor, ME, USA). Mice between 8 and 12 weeks of age were used for the experiments. All the experiments were performed in accordance with the guidelines of the animal ethics committee of the University of Tsukuba Animal Research Center (permission number: #17-145). All works using human samples were approved by the institutional review board of the University of Tsukuba (permission number: H28-1).

### 2.2. Cell Lines

B16F10 melanoma cells, Lewis lung carcinoma (LLC) cells, MB49 bladder cancer cells, and MC38 colon cancer cells were cultivated in DMEM (Thermo Fisher, Waltham, MA, USA) supplemented with 10% FBS, 1% penicillin-streptomycin (FUJIFILM Wako, Osaka, Japan, 100 mM sodium pyruvate (Wako), 1% MEM nonessential amino acids solution (Wako), and 100 mM L-alanyl-L-glutamine (Wako) at 37 °C under 5% CO_2_.

### 2.3. Murine Tumor Model

We intradermally injected 1 × 10^6^ of MB49 and LLC, 3 × 10^5^ cells of B16F10, or 2 × 10^6^ cells of MC38 cells into the backs of the mice. After inoculation of the mice, the diameters of the tumors were measured with a caliper and the tumor volume was determined according to the following formula: tumor volume (mm^3^) = (length) × (width)^2^ × 0.5. Topical 5% IMQ cream (Beselna Cream™) was kindly gifted from Mochida Pharmaceutical Co., Ltd., and 83 mg of this reagent was topically applied to the tumor on every other day from day 2 to day 18. Anti-PD-1 antibody (clone: 4H2) was kindly gifted from Ono Pharmaceutical. The mice were given intraperitoneal injections of anti-PD-1 antibody (100 μg/mouse) every 4 days from day 4 to day 16 unless otherwise indicated in the figure legends.

### 2.4. Histopathologic Analyses

Tissue blocks were fixed in 10% formalin. After paraffin embedding, 3-μm sections were subjected to staining. For the cell number counts, 5 randomly selected sites at 400× magnification (high-power field) were evaluated by use of light microscopy. For immunohistochemistry of CD4, CD8, or FoxP3, the sections were deparaffinized in xylene and rehydrated before antigen retrieval by boiling in citrate buffer (0.01 M citrate containing 0.5% Tween 20, pH 6.0). The sections were incubated in 10% bovine serum albumin (BSA) in PBS at room temperature for 1 h and then stained with rat anti-CD4 antibody (4SM95, 1:500 dilution; eBioscience, San Diego, CA, USA), anti-CD8 antibody (4SM15, 1:500 dilution; eBioscience), or anti-FoxP3 antibody (FJK-16s, 1:200 dilution; Invitrogen, Waltham, MA, USA) overnight at 4 °C, followed by biotinylated anti-rat IgG antibody (1:500; Vector Laboratories, Burlingame, CA, USA) and Vectastain ABC reagent (Vector Laboratories) at room temperature for 60 min and 30 min, respectively. Finally, the sections were stained by the use of a DAB Peroxidase Substrate Kit (Vector Laboratories) before imaging. For detection of apoptotic cells, a TumorTACS in Situ Apoptosis Detection Kit (Trevigen, Gaithersburg, MD, USA) was used according to the manufacturer’s instructions.

### 2.5. In Vitro Assay

Splenocytes or MC38 cells were seeded at 1.5 × 10^6^ cells/well (splenocytes) or 1.0 × 10^6^ cells/well (MC38) in a 6-well plate in complete DMEM with 4 μg/mL of IMQ (InvivoGen, San Diego, CA, USA) or deionized distilled water as the control. After stimulation for the indicated time, the cells were used for counting living cell numbers, flow cytometric analyses, or analyses of the quantitative reverse transcription-polymerase chain reaction (qRT-PCR).

For the JAK inhibition assay, splenocytes were cultured with tofacitinib (Selleck, Houston, TX, USA) at a final concentration of 300 μmol/L or with PBS as the control. For the nuclear factor-kappa B (NF-κB) inhibition assay, splenocytes were cultured with BMS-345541 (Chemscene, Monmouth Junction, NJ, USA) at a final concentration of 2.5 μmol/L, and DMSO as the control. After treatment for 24 h, the cells were used for flow cytometric analyses.

### 2.6. Cell Isolation

For cell isolation, whole tumors were minced with scissors and digested in complete medium with 2 mg/mL crude collagenase (FUJIFILM Wako) and 2 KU/mL deoxyribonuclease I (Sigma, St. Louis, MI, USA) with a GentleMACS tissue processor. The digested tissue was cultured at 37 °C for 30 min to prepare the tumor cell suspension. For cell isolation from the regional LNs of the inoculated tumor, inguinal LNs were pressed down with a syringe plunger to make a single-cell suspension. For isolation of human peripheral blood mononuclear cells (PBMCs), blood samples were collected from patients with skin tumors, and PBMCs were isolated from the blood by means of Ficoll density gradient centrifugation according to the manufacturer’s instructions (density 1.077; GE Healthcare, Chicago, IL, USA).

### 2.7. Flow Cytometric Analysis

Isolated cells were incubated in FACS staining buffer (PBS containing 5% BSA and 0.01% NaN3) with FcR Blocking Reagent (Miltenyi Biotec, Bergisch Gladbach, Germany). The cells were then stained with antibodies. The following antibodies were purchased from BioLegend: anti-CD4 (GK1.5), anti-CD8α (53-6.7), anti-CD11b (M1/70), anti-CD11c (N418), anti-CD19 (6D5), anti-CD45.2 (104), anti-CD69 (H1.2F3), anti-CD80 (16-10A1), anti-CD86 (GL-1), anti-PD-L2 (TY25), anti-PD-1 (29F.1A12), anti-I-A/I-E (2G9), anti-CD38 (90), anti-TRAIL (tumor necrosis factor-related apoptosis-inducing ligand) (N2B2), anti-TLR7 (Rabbit polyclonal), anti-granzyme B (QA16A02), anti-perforin (S16009A), and anti-IFN-γ (XMG1.2) mAbs for murine cell staining; anti-CD3ε (UCHT1), anti-CD4 (OKT4), anti-CD8α (RPA-T8), anti-CD11c (3.9), anti-CD19 (HIB19), anti-CD45 (HI30), anti-CD80 (2D10), anti-CD86 (clone: IT2.2), anti-PD-L2 (MIH18), anti-PD-L1 (29E.2A3), anti-PD-1 (EH12.2H7), and anti-HLA-DR, DP, DQ (Tu39) mAbs for human cell staining; and anti-CD11b (M1/70) mAb for both murine and human cell staining. Anti-CD3ε (145-2C11) and anti-PD-L1 (MIH5) mAbs for murine cell staining were purchased from eBioscience and BD Biosciences, respectively. Dead cells were stained by the addition of the 7-AAD Viability Staining Solution (eBioscience).

For intracellular TLR7, granzyme B, and perforin staining, the cells were stained with a Zombie Fixable Viability Kit (BioLegend, San Diego, CA, USA) to detect the dead cells. Intracellular molecules were stained using a BD Cytofix/Cytoperm™ Fixation/Permeabilization Solution Kit (Becton, Dickinson and Company, Franklin Lakes, NZ, USA) for TLR7, or a True-Nuclear™ Transcription Factor Buffer Set (BioLegend) for granzyme B and perforin in accordance with the manufacturer’s instructions.

For intracellular IFN-γ staining, the cells were stimulated with 25 ng/mL PMA and 1 µg/mL ionomycin in RPMI 1640 medium supplemented with 10% fetal bovine serum, 2 mM L-glutamine, 100 U/mL penicillin, 100 µg/mL streptomycin, and 0.2 mM 2-mercaptoethanol with monensin (BioLegend). A Zombie Fixable Viability Kit (BioLegend) was used for staining the dead cells. Intracellular IFN-γ was stained using a True-Nuclear™ Transcription Factor Buffer Set (BioLegend) in accordance with the manufacturer’s instructions.

Flow cytometry was performed on a Beckman Coulter Gallios instrument (Beckman Coulter, Brea, CA, USA), and the data were analyzed using Kaluza Flow Analysis software (Beckman Coulter).

### 2.8. qRT-PCR

Total RNA was isolated by the use of Trizol Reagent (Invitrogen). Analyses of qRT-PCR were performed on the QuantStudio™ 5 Real-Time PCR System (Applied Biosystems) with PrimeTime^®^ Gene Expression Master Mix and Prime Tim qPCR predesigned primers (Integrated DNA Technologies). The mRNA level of each gene was normalized to that of glyceraldehyde-3-phosphate dehydrogenase (GAPDH). The primers used were as follows: *Ccl2*: Mm.PT.58.42151692, *Tnf-α*: Mm.PT.58.12575861; *Il-2*: Mm.PT.58.11478202; *Il-6*: Mm.PT.58.10005566; *Il-15*: Mm.PT.58.28815139.

### 2.9. Measurement of Cytokines

Cytokine concentrations in splenocyte culture supernatants were analyzed with the Mouse T Helper Cytokine Panel (BioLegend) according to the manufacturer’s instructions. Bead fluorescence was measured using the Beckman Coulter Gallios instrument (Beckman Coulter) and analyzed using the Biolegend LEGENDplex^TM^ data analysis software.

### 2.10. Killing Assay

Target tumor cells were labeled with calcein-AM fluorescent dye (Dojindo, Kumamoto, Japan). Cells from lymph nodes (1.28 × 10^6^) were cocultured with target tumor cells (1.0 × 10^4^) in calcium and magnesium-free Hank’s balanced salt solution with 5% fetal bovine serum in 96-well plates for 1.0 h at 37 °C. Lysis buffer (50 mM sodium borate and 0.1% Triton X in distilled water) was used to induce maximum target cell lysis. The fluorescence release was measured on a fluorimeter (excitation/emission = 485 nm/535 nm). Percentage lysis was determined in the following manner: (experimental release—spontaneous release)/(maximum release—spontaneous release) × 100.

### 2.11. Statistical Analyses

Statistical analyses were performed using the Mann–Whitney U test for analyses of the mouse experiments. For the human PBMC analyses, the Wilcoxon matched-pairs signed-rank test was used. Throughout the analyses, probability values < 0.05 were considered significant. The statistical tests were 2-sided and carried out using Prism version 9 (GraphPad Software, San Diego, CA, USA).

## 3. Results

### 3.1. Topical IMQ Exerts an Antitumor Effect through Enhancement of the Immune Response to MC38 Colon Cancer

**Figure 1:**
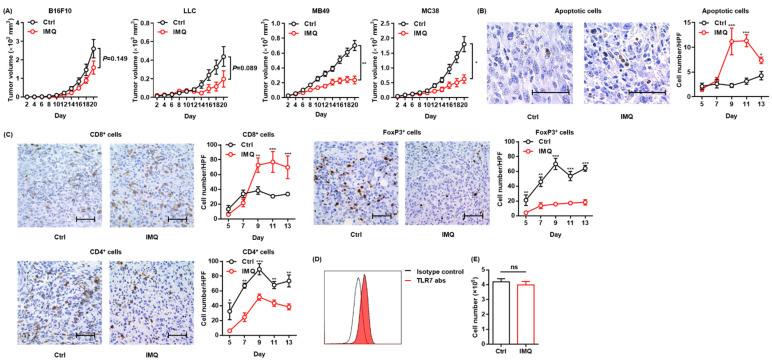
Topical IMQ exerts an antitumor effect through the enhancement of the immune response to MC38 colon cancer. (**A**) Time course of the tumor volume after 3 × 10^5^ B16F10 melanoma cells, 1 × 10^6^ LLC cells, MB49 bladder cancer cells, or 2 × 10^6^ MC38 colon cancer cells were intradermally inoculated into the backs of wildtype mice. IMQ was topically applied to the back over the tumor every other day from day 2 to day 18, and untreated mice were used as the control (*n* = 10 [B16F10] and 6 [LLC, MB49, and MC38] in each group). (**B**,**C**) Representative histopathology of apoptosis staining (**B**) or immunohistochemical staining (**C**) at day 9, and the number of apoptotic tumor cells (**B**) and the number of CD8^+^ T cells, CD4^+^ T cells, and Foxp3^+^ cells (**C**) in the tumor at each indicated day after the inoculation of MC38 cells into wildtype mice, quantified in 5 randomly selected high-power field (HPF) images per mouse (*n* = 6 in each group of days 5 and 7, and *n* = 7 in each group of days 9, 11, and 13, respectively. Bar = 50 μm). (**D**) Representative flow cytometric analysis of TLR7 expression in MC38 in vitro. (**E**) The number of living MC38 cells stimulated with IMQ or the control for 3 days in vitro *(n* = 6 in each group). Ctrl: control, IMQ: imiquimod, HPF: high-power (400×) field, abs: antibodies, ns: not significant. Error bars indicate ±1 SEM; * *p* < 0.05, ** *p* < 0.01, *** *p* < 0.001.

To confirm the antitumor effect of topical IMQ, we intradermally inoculated B16F10 melanoma cells into mice and examined the effect of topical IMQ on tumor growth. However, unlike in previous studies showing an antitumor effect of topical IMQ on B16F10 melanoma [13,26], in our experiments, topical IMQ did not significantly inhibit tumor growth (Figure 1A). Topical IMQ did not also significantly inhibit the tumor growth of LLC cells, although there was a tendency toward inhibiting the tumor growth (Figure 1A). Both B16F10 and LLC are known to be low-immunogenic tumors [27,28]. Meanwhile, topical IMQ significantly suppressed the tumor growth of both MB49 bladder cancer cells and MC38 colon cancer cells, both of which have been reported to show high immunogenicity [27,28,29] (Figure 1A). To clarify the mechanism of the antitumor effect of IMQ in our study, we evaluated apoptotic tumor cells by use of a TUNEL assay and T-cell infiltration by immunohistochemical studies in the MC38 tumor model. We found that the number of apoptotic tumor cells increased (Figure 1B) along with the increased number of CD8^+^ T cells within the tumors, whereas the number of CD4^+^ T cells and FoxP3^+^ regulatory T cells (Tregs) decreased with topical IMQ (Figure 1C). Previous studies demonstrated that TLR 7 is expressed in some tumor cells and that IMQ may directly induce apoptosis of the tumor cells through TLR 7 ligation [24]. We confirmed the expression of TLR7 in MC38 (Figure 1D). To assess the direct antitumor effect of IMQ on MC38, we counted the number of viable MC38 cells after the addition of IMQ in vitro. However, the number of viable MC38 was not affected by the addition of IMQ for 3-day culture in vitro, suggesting that IMQ did not directly affect the cell growth of MC38 (Figure 1E). These results suggest that topical IMQ exerts its antitumor effect by enhancing the immune response to MC38 in the murine tumor model.

### 3.2. IMQ Activates Myeloid Cells, Leading to Upregulation of Costimulatory Molecules and MHC Class II

Since previous studies showed that the TLR 7 agonist activated myeloid cells [4,30,31], we examined the expression of TLR 7 in myeloid cells. We classified myeloid cells into 3 subsets as follows: MHC class II^+^ CD11b^+^ CD11c^−^ cells (macrophages in tissues or monocytes in human blood), MHC class II^+^ CD11b^+^ CD11c^+^ cells (CD11b^+^ DCs), and MHC class II^+^ CD11b^−^ CD11c^+^ cells (CD11b^−^ DCs) (Figure S1) and confirmed that all the subsets of myeloid cells in murine spleen expressed TLR 7 (Figure S2).

**Figure 2:**
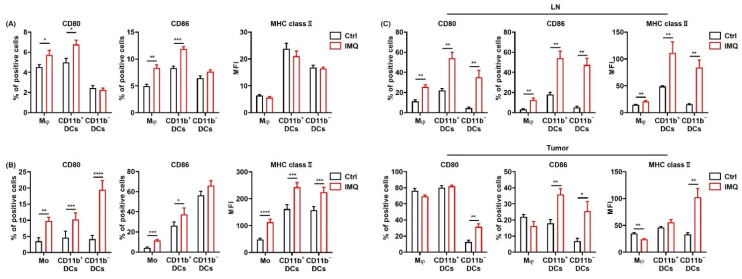
IMQ activates myeloid cells, leading to the upregulation of costimulatory molecules and MHC class II. (**A**) Flow cytometric analysis of CD80, CD86, and MHC class II expression in myeloid cells of the murine spleen (**A**) and human blood (**B**) stimulated with IMQ for 24 h *(n* = 8 (**A**) and *n* = 18 (**B**) in each group, respectively). (**C**) Flow cytometric analysis of CD80, CD86, and MHC class II expression of myeloid cells in the lymph nodes and tumors 5 days after tumor inoculation with topical IMQ application at day 4 (*n* = 6 in each group). Ctrl: control, IMQ: imiquimod, Mφ: macrophages, Mo: monocytes, DCs: dendritic cells, LNs: lymph nodes. Error bars indicate ±1 SEM; * *p* < 0.05, ** *p* < 0.01, *** *p* < 0.001, **** *p* < 0.0001.

Next, we examined the expressions of CD80, 86, and MHC class II, which are known as the activation markers of myeloid cells, in macrophages/monocytes and dendritic cells (DCs) after stimulation by IMQ in vitro. We found that CD80 and CD86 expression in macrophages and CD11b^+^ DCs in murine spleen were significantly upregulated by IMQ stimulation, whereas MHC class II expression was comparable (Figure 2A). Moreover, TRAIL expression in all the myeloid cell subsets was significantly upregulated (Figure S3).

We also analyzed the expression of these molecules in human PBMCs. Similarly, CD80 and MHC class II expression in all the subsets and CD86 expression in monocytes and CD11b^+^ DCs were upregulated by in vitro IMQ stimulation (Figure 2B).

To investigate the effect of IMQ in vivo, we applied IMQ cream after inoculation of MC38 cells into the mice and assessed the expression of the activation markers on the myeloid cells in the LNs and tumors. The results showed that the CD80, 86, and MHC class II expression of all the subsets in the LNs was significantly upregulated by topical IMQ (Figure 2C). In addition, CD80 expression in CD11b^−^ DCs; CD86 expression in CD11b^+^ DCs, and CD11b^−^ DCs; and MHC class II expression in the macrophages, and CD11b^−^ DCs in the tumors was significantly upregulated by topical IMQ (Figure 2C). These results suggest that IMQ activates myeloid cells, leading to upregulation of costimulatory molecules and MHC class II.

### 3.3. Antitumor Effect of IMQ Is Dependent on Adaptive Immunity

**Figure 3:**
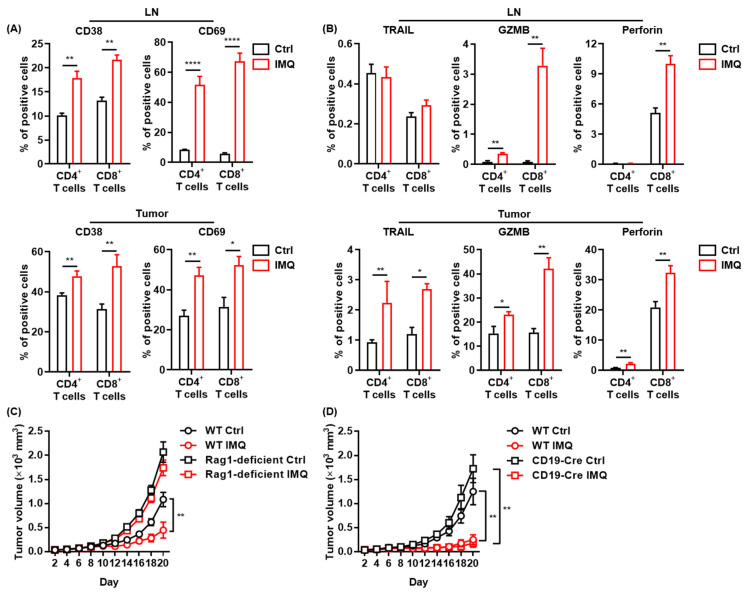
The antitumor effect of IMQ is dependent on adaptive immunity. (**A**,**B**) Flow cytometric analysis of expression of CD38, CD69 (**A**), TRAIL, GZMB, and perforin (**B**) in CD4+ T cells and CD8+ T cells in LNs and tumors 5 (CD69) or 7 (CD38, TRAIL, GZMB, and perforin) days after tumor inoculation with IMQ application at day 4 (*n* = 12 [CD69] or 6 [CD38, TRAIL, GZMB, and perforin] in each group). (**C**,**D**) Time course of the tumor volume after 2 × 10^6^ MC38 colon cancer cells were intradermally inoculated into the backs of Rag1-deficient mice (**B**) or CD19-Cre transgenic mice (**C**) with topical IMQ application or no treatment (*n* = 11 in Rag1-deficient mice with IMQ application and *n* = 12 in Rag1-deficient mice with no treatment, homozygous CD19-Cre transgenic mice with topical IMQ application or no treatment). WT: wildtype, Ctrl: control, IMQ: imiquimod, LNs: lymph nodes., TRAIL: tumor necrosis factor-related apoptosis-inducing ligand, GZMB: granzyme B. Error bars indicate ±1 SEM; * *p* < 0.05, ** *p* < 0.01, **** *p* < 0.0001.

Since IMQ upregulated the expression of costimulatory molecules in myeloid cells, which play essential roles in T-cell activation [32], we assessed T-cell activation after topical IMQ administration in the murine tumor model. We found that expression of CD38 and CD69, activation markers of T cells, in both CD4^+^ T cells and CD8^+^ T cells in the LNs and tumors was significantly upregulated by topical IMQ (Figure 3A). Moreover, IMQ enhanced the expression of TRAIL, granzyme B, and perforin in both CD4^+^ T cells and CD8^+^ T cells in tumors (Figure 3B). IMQ also upregulated granzyme B expression in both CD4^+^ T cells and CD8^+^ T cells and perforin expression in CD8^+^ T cells in the LNs (Figure 3B). These results suggest that myeloid cells activated by topical IMQ stimulation may induce an antitumor effect by T-cell activation.

To clarify the role of adaptive immunity in the antitumor effect of IMQ, we assessed the IMQ-induced antitumor effect in Rag1-deficient mice, which lack T and B cells. The results showed that topical IMQ failed to inhibit the tumor growth in Rag1-deficient mice (Figure 3C), indicating that the antitumor effect of IMQ is dependent on adaptive immunity. Next, we examined the effect of topical IMQ in homozygous CD19-Cre transgenic mice, which show significantly impaired B cell activation [33]. We found that the IMQ-induced antitumor effect in homozygous CD19-Cre transgenic mice was comparable to that in wildtype mice (Figure 3D). These results indicate that B cells might not play a major role in the IMQ-induced antitumor effect.

### 3.4. The IMQ-Induced Antitumor Effect Was Dependent on Increased IFN-γ Expression

**Figure 4:**
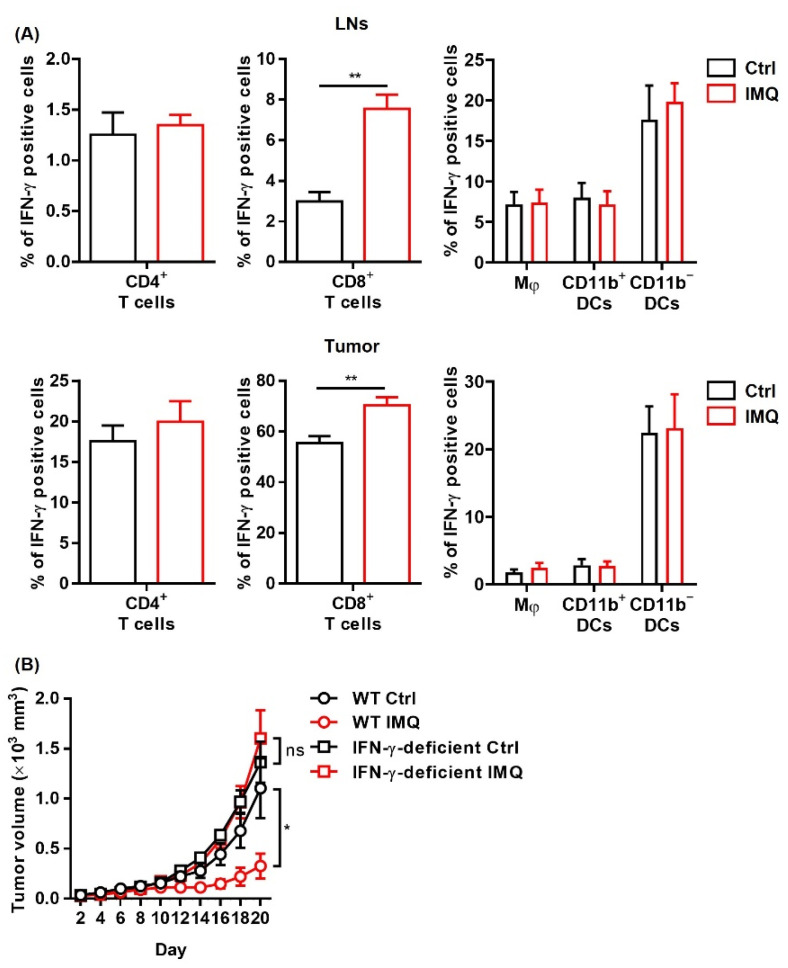
The IMQ-induced antitumor effect is dependent on IFN-γ. (**A**,**B**) Flow cytometric analysis of IFN-γ expression in CD4^+^ T cells, CD8^+^ T cells, Mφ, CD11b^+^ DC, and CD11b^−^ DC in the lymph nodes (**A**) and tumors (**B**) 8 days after tumor inoculation with IMQ application at day 7 (*n* = 5 in each group). (**C**) Time course of the tumor volume after 2 × 10^6^ MC38 colon cancer cells were intradermally inoculated into the backs of wildtype or IFN-γ-deficient mice with topical IMQ application or no treatment (*n* = 8 in each group). WT: wildtype, Ctrl: control, IMQ: imiquimod, Mφ: macrophages, DCs: dendritic cells, LNs: lymph nodes, IFN-γ: interferon γ, ns: not significant. Error bars indicate ±1 SEM; * *p* < 0.05, ** *p* < 0.01.

Since IFN-γ exerts a potent antitumor effect [34], we investigated IFN-γ expression after stimulation of topical IMQ in the murine tumor model. We found that topical IMQ significantly upregulated IFN-γ expression in CD8^+^ T cells in both the LNs and the tumors, whereas IFN-γ expression in other immune cells including CD4^+^ T cells, macrophages, and DCs were not greatly affected (Figure 4A). To investigate the role of IFN-γ in the IMQ-induced antitumor effect, we tested IFN-γ deficient mice. We found that the IMQ-induced antitumor effect was abolished in the IFN-γ deficient mice (Figure 4B), indicating that IFN-γ is indispensable to the IMQ-induced antitumor effect.

### 3.5. IMQ Upregulates Expression of PD-1/PD-L1/PD-L2 in Immune Cells

**Figure 5:**
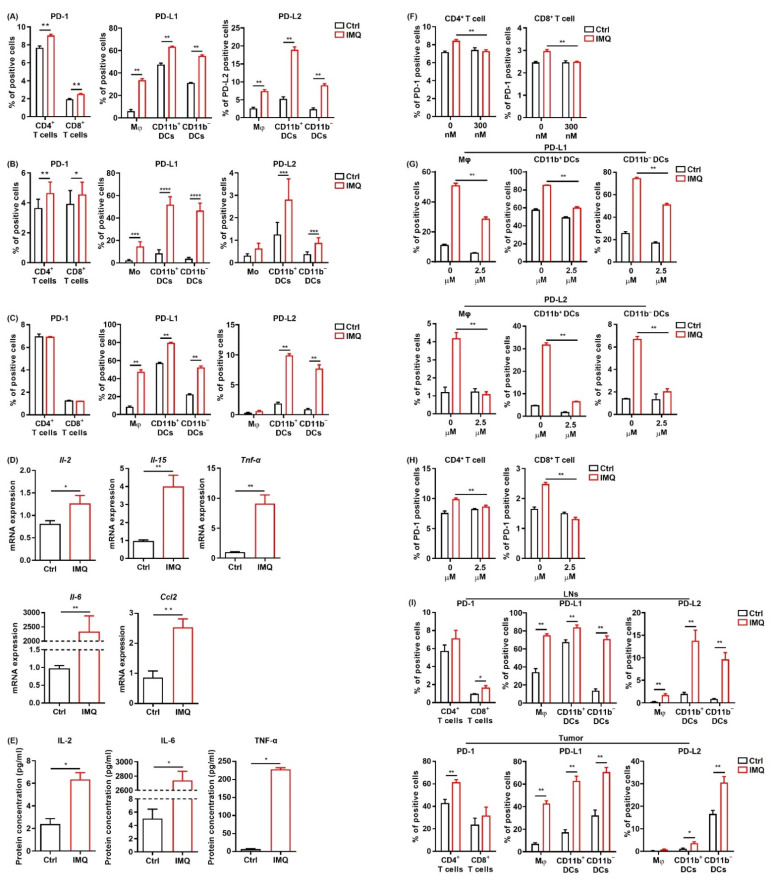
IMQ upregulates the expression of PD-1/PD-L1/PD-L2 in immune cells. (**A**–**C**) Flow cytometric analysis of PD-1 expression in CD4^+^ T cells and CD8^+^ T cells, and PD-L1 and PD-L2 expression in myeloid cells of the murine spleen (**A**,**C**) and human blood (**B**) stimulated with IMQ for 24 h (**A**,**B**) or 6 h (**C**) (*n* = 6 (**A**,**C**) and *n* = 18 (**B**) in each group, respectively). (**D**) Quantitative RT-PCR analysis of cytokine expression of murine splenocytes stimulated with IMQ for 6 h (*n* = 6 in each group). (**E**) Cytokine concentrations in culture supernatants of splenocytes 24 h after IMQ stimulation (*n* = 4 in each group). (**F**) Flow cytometric analysis of PD-1 in CD4^+^ T cells and CD8^+^ T cells stimulated with IMQ in the absence or presence of 300 μM of tofaicitinib for 24 h (*n* = 6 in each group). (**G**,**H**) Flow cytometric analysis of PD-L1 and PD-L2 expression in myeloid cells (**G**) and of PD-1 expression in CD4^+^ T cells and CD8^+^ T cells (**H**) stimulated with IMQ in the presence of 2.5 μM of BMS-345541 for 24 h (*n* = 6 in each group). (**I**) Flow cytometric analysis of PD-1 expression in CD4^+^ T cells and CD8^+^ T cells and of PD-L1 and PD-L2 expression in myeloid cells in the lymph nodes and tumor 5 days after tumor inoculation with IMQ application at day 4 *(n* = 6 in each group). Ctrl: control, IMQ: imiquimod, Mφ: macrophages, Mo: monocytes, DCs: dendritic cells, LNs: lymph nodes. Error bars indicate ±1 SEM; * *p* < 0.05, ** *p* < 0.01, *** *p* < 0.001, **** *p* < 0.0001.

Next, we investigated the change in PD-1 expression in T cells and PD-L1/L2 expression in the myeloid cells of murine splenocytes after IMQ stimulation in vitro. We found that PD-1 expression in both CD4^+^ T cells and CD8^+^ T cells and PD-L1/L2 expression in all the myeloid cell subsets were significantly upregulated 24 h after IMQ stimulation (Figure 5A). In contrast, PD-L1/L2 expression in MC38 cells was not changed after IMQ stimulation in vitro (Figure S4). Next, we analyzed the expression of PD-1 and PD-L1/L2 in human PBMCs after IMQ stimulation. Similarly, PD-1 expression in CD4^+^ T cells and CD8^+^ T cells, PD-L1 expression in all the myeloid cell subsets, and PD-L2 expression in CD11b^+^ DCs and C11b^−^ DCs were upregulated 24 h after IMQ stimulation (Figure 5B). Next, we examined the PD-1 and PD-L1/L2 expression of murine splenocytes 6 h after IMQ stimulation. We found that PD-1 expression in T cells had yet to be increased 6 h after the stimulation, whereas PD-L1 expression in all the myeloid cell subsets and PD-L2 expression in CD11b^+^ DCs and CD11b^−^ DCs were already upregulated (Figure 5C). Because T cells show low expression of TLR 7 [35,36], the IMQ-induced upregulation of PD-1 in T cells 24 h after the stimulation may be the consequence of increased cytokine production of other cells such as myeloid cells and B cells rather than of direct stimulation by IMQ as a TLR 7 ligand. Indeed, the mRNA expressions of Il2, Il15, TNFα, Il6, and Ccl2 in the splenocytes were increased 6 h after the stimulation (Figure 5D). Since a previous study revealed that enhanced production of CCL2 from tumor tissue may be involved in TLR7/8-induced antitumor effects [6], we also evaluated Ccl2 expression in MC38 cells in the presence of IMQ and found that IMQ significantly upregulated the expression of Ccl2 in MC38 cells (Figure S5). The concentrations of IL-2, IL-6, and TNF-α in the culture supernatant 24 h after stimulation of IMQ were also significantly increased (Figure 5E). In addition, we found that the IMQ-induced upregulation of PD-1 in T-cells was abolished by the JAK inhibitor, tofacitinib (Figure 5F), suggesting that JAK signaling plays important roles in the IMQ-induced upregulation of PD-1 in T cells.

To elucidate the role of NF-κB, which is the signal transducer of several cytokines and TLR signals, in IMQ-induced PD-1 and PD-L1/L2 upregulation, we used a highly selective inhibitor of IκB kinase, BMS-345541. This BMS-345541 significantly suppressed the IMQ-induced upregulation of PD-L1/L2 expression in all the myeloid cell subsets 24 h after the stimulation (Figure 5G), suggesting that IMQ-induced upregulation of PD-L1/L2 expression in myeloid cells is largely dependent on NF-κB signaling. We also found that IMQ-induced upregulation of PD-1 in T cells 24 h after the stimulation was significantly suppressed by BMS-345541 (Figure 5H), suggesting that BMS-345541 suppresses IMQ-induced upregulation of PD-1 expression in T cells through inhibition of cytokine production from other immune cells. Taken together, these results indicate that NF-κB plays vital roles in the IMQ-induced upregulation of PD-1 and PD-L1/L2 expression.

Next, we evaluated PD-1 and PD-L1/L2 expression after stimulation of topical IMQ in the murine tumor model. Consistent with the in vitro findings, topical IMQ significantly upregulated PD-1 expression in CD4^+^ T cells and CD8^+^ T cells in the LNs and tumors, respectively (Figure 5I). In addition, PD-L1/PD-L2 expression in most of the myeloid cell subsets in both the LNs and the tumors was significantly upregulated (Figure 5I). On the other hand, PD-L1/L2 expression in MC38 cells was unchanged (Figure S6). Collectively, these results suggest that IMQ upregulates the expression of PD-1 and PD-L1/L2 in immune cells both in vitro and in vivo.

### 3.6. IFN-γ Is Not Involved in the IMQ-Induced Upregulation of PD-L1 Expression

**Figure 6:**
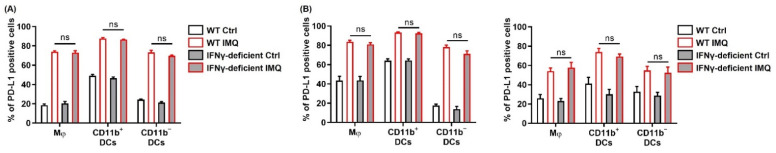
IFN-γ is not involved in the IMQ-induced upregulation of PD-L1 expression. (**A**) Flow cytometric analysis of PD-L1 expression in myeloid cells of the spleens of wildtype or IFN-γ-deficient mice stimulated with IMQ for 24 h (*n* = 6 in each group). (**B**) Flow cytometric analysis of PD-L1 expression in the myeloid cells of the lymph nodes and tumors of wildtype or IFN-γ-deficient mice 5 days after tumor inoculation with IMQ application at day 4 (*n* = 8 in wildtype mice with IMQ application or no treatment or IFN-γ-deficient mice with no treatment, *n* = 7 in IFN-γ-deficient mice with IMQ treatment). WT: wildtype, Ctrl: control, IMQ: imiquimod, Mφ: macrophages, DCs: dendritic cells, LNs: lymph nodes, IFN-γ: interferon γ, ns: not significant. Error bars indicate ±1 SEM.

Previous studies have demonstrated that IFN-γ is the most prominent factor to upregulate PD-L1 expression, although PD-L2 is not upregulated by IFN-γ [37,38]. However, the PD-L1 expression in the myeloid cells of the murine spleen after IMQ stimulation in vitro was comparable between the wildtype mice and the IFN-γ-deficient mice (Figure 6A). Similarly, the PD-L1 expression in the myeloid cells of both the LNs and the tumors after topical IMQ stimulation in the tumor murine model were also comparable between the wildtype mice and the IFN-γ-deficient mice (Figure 6B). These results indicate that IFN-γ is not involved in the IMQ-induced upregulation of PD-L1 expression.

### 3.7. Potent Antitumor Effect of Combination Therapy of Topical IMQ Plus Anti-PD-1 Antibody

**Figure 7:**
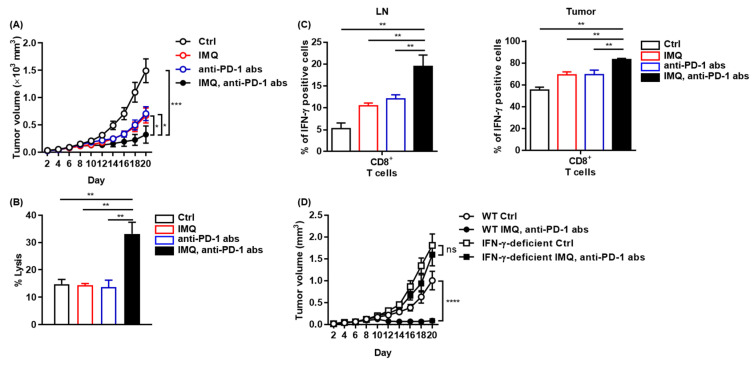
Potent antitumor effect of combination therapy of topical IMQ with anti-PD-1 antibody. (**A**) Time course of the tumor volume after 2 × 10^6^ MC38 colon cancer cells were intradermally inoculated into the backs of wildtype mice with topical IMQ application, anti-PD-1 antibody treatment, topical IMQ plus anti-PD-1 antibody treatment, or no treatment (*n* = 12 in each group). (**B**) Percentage killing of MC38 colon cancer cells by immune cells from LNs 9 days after the inoculation of MC38 colon cancer cells into wildtype mice with topical IMQ application, anti-PD-1 antibody treatment, topical IMQ plus anti-PD-1 antibody treatment, or no treatment (*n* = 6 in each group). (**C**) Flow cytometric analysis of IFN-γ expression in CD8^+^ T cells in lymph nodes and tumors 10 days after tumor inoculation with topical IMQ application and anti-PD-1 antibody administration at day 8 (*n* = 5 in each group). (**D**) Time course of the tumor volume after 2 × 10^6^ MC38 colon cancer cells were intradermally inoculated into the backs of wildtype mice with topical IMQ monotherapy, anti-PD-1 antibody monotherapy, topical IMQ application plus anti-PD-1 antibody treatment, or no treatment (*n* = 11 in wildtype mice with no treatment, *n* = 10 in wildtype mice with IMQ application plus anti-PD-1 antibody treatment or IFN-γ-deficient mice with IMQ application with anti-PD-1 antibody treatment or no treatment). WT: wildtype, Ctrl: control, IMQ: imiquimod, LNs: lymph nodes, IFN-γ: interferon γ, abs: antibodies, ns: not significant. Error bars indicate ±1 SEM; * *p* < 0.05, ** *p* < 0.01, *** *p* < 0.001, **** *p* < 0.0001.

We found that IMQ upregulated PD-1 expression in T cells and PD-L1/L2 expression in myeloid cells both in vitro and in vivo, indicating that IMQ induces not only T-cell activation but also T-cell exhaustion, which may dampen IMQ-induced antitumor immunity. Next, we assessed combination therapy of topical IMQ and anti-PD-1 antibody. We found that the combination therapy significantly suppressed the tumor growth of MC38 as compared with each monotherapy (Figure 7A), although neither anti-PD-1 antibody monotherapy nor the combination therapy was effective for B16F10 melanoma (Figure S7). Consistent with marked suppression of MC38 tumor growth, the combination therapy enhanced immune cell-mediated cytotoxicity against MC38 relative to the respective monotherapy and no treatment (Figure 7B). In addition, the population of IFN-γ-positive CD8^+^ T cells in both the LNs and the tumors of MC38 in mice treated with the combination therapy was even higher than that in mice treated with topical IMQ monotherapy (Figure 7C). By contrast, the antitumor effect of the combination therapy was abrogated when tested in IFN-γ-deficient mice, indicating that the antitumor effects of both topical IMQ and anti-PD-1 antibody are dependent on IFN-γ (Figure 7D). Collectively, these results suggest that the combination therapy of topical IMQ plus anti-PD-1 antibody shows a significantly potent antitumor effect through enhancement of IFN-γ production.

## 4. Discussion

In our study, topical IMQ did not exert a significant antitumor effect against B16F10 or LLC. In contrast, IMQ significantly suppressed the tumor growth of MB49 and MC38. Given that both MB49 and MC38 have been reported to show high immunogenicity whereas B16F10 and LLC are poorly immunogenic tumors [27,28,29], the antitumor effect of topical IMQ depends on an enhanced immunologic response to the tumor. Although some studies have shown a significant antitumor effect of topical IMQ against B16F10 melanoma [13,26], other studies have shown a poor antitumor effect of IMQ against B16F10 melanoma, as in our study [39,40]. The difference may be due to a discrepancy in the methods, including the inoculated tumor cell number, amount of applied IMQ, and schedule of IMQ application in each study. Similarly, the antitumor effect of anti-PD-1/PD-L1 antibodies for B16F10 varies between studies [15,41,42,43]. It has been reported that C57BL/6 mice derived from two different mouse facilities exhibited differences in the immune response to the tumor, resulting in different growth of a melanoma cell line, which was associated with different commensal microbes in these mice [44]. Therefore, the difference might be due to a discrepancy of environmental factors of each facility such as commensal microbes. Since the genomic and transcriptional profiles differ between cancer cell lines and human tumor samples, cancer cell lines do not always recapitulate human tumor cells [45]. Although some melanoma cell lines show gene expression patterns similar to those of human melanoma, the expressions of many genes related to immune function were shown to be significantly different [45]. In addition, the immunogenicity of human melanoma is also largely distinct in each case [46]. Indeed, cutaneous melanoma shows significantly higher immunogenicity than do acral lentiginous melanoma and mucosal melanoma, which may be associated with a higher tumor mutation burden in cutaneous melanoma [47,48]. Therefore, although topical IMQ did not significantly affect the tumor growth of B16F10 melanoma cell lines in our study, it may exert an antitumor response to human melanoma depending on the immunogenicity of each case.

Although the exact mechanism of the antitumor effect induced by the TLR 7/8 ligands is largely unknown, previous animal and human studies have suggested that a variety of cytokines induced by TLR 7/8 ligand engagement are involved in the effect [6,7,9,10]. Aspord et al. reported that IMQ inhibited melanoma cell growth through upregulation of the type I IFN response genes in a humanized mouse model [9]. Wang et al. demonstrated that loxoribine, one of the TLR 7 ligands, could inhibit tumor growth in both colon cancer and lung cancer xenograft models through increased IL-6 production from DCs [10]. Haung et al. showed that tumor-infiltrating T cells from squamous cell carcinoma treated with topical IMQ in humans produced more IFN-γ, granzyme, and perforin than those from untreated tumors [7]. In addition, Singh et al. reported that intratumoral administration of 3M-052, a tissue-retained TLR 7/8 agonist, suppressed tumor growth in a murine tumor model using B16F10 melanoma cells. In their model, enhanced production of CCL2 in the tumor tissue induced by 3M-052 played important roles for the antitumor effect, whereas both type I IFNs and IFN-γ contributed only partially to the antitumor effect [6]. Wu et al. reported that upregulated expression of TRAIL may be involved in the IMQ-induced antitumor effect [11]. Therefore, the mechanisms of the TLR 7/8 agonist-induced antitumor effect may be diverse in a context-dependent manner. Although IFN-γ was crucial in the IMQ-induced antitumor effect in our study, our study did not exclude the importance of other molecules that previous studies suggested. Indeed, we found significant upregulation of CCL2 in splenocytes and MC38 cells, IL-6 in splenocytes, and TRAIL in myeloid cells of the spleen as well as of T cells in the tumors by IMQ stimulation.

In our murine tumor model, topical IMQ failed to inhibit the tumor growth in Rag1-deficient mice, suggesting that the IMQ-induced antitumor effect is dependent on adaptive immunity. In contrast, the IMQ-induced antitumor effect in homozygous CD19-Cre transgenic mice was comparable to that in wildtype mice, indicating that B cells might not play a major role in the IMQ-induced antitumor effect. However, this finding may be insufficient to completely exclude the involvement of B cells in the IMQ-induced antitumor effect since B-2 cells are preserved in the homozygous CD19-Cre transgenic mice, although there is a defect in B-1 cells and marginal zone B cells [33]. Thus, further studies are required for clarifying the role of B cells in the IMQ-induced antitumor effect. In our study, we also showed antitumor effect is dependent on IFN-γ. IFN-γ is known to play vital roles in antitumor immunity by coordinating several biologic responses primarily involved in the establishment of adaptive immunity toward the Th1-mediated immune response [49]. IFN-γ not only enhances CD8^+^ T cell motility and killing of tumor cells but also activates antigen-presenting cells [50]. In our study, IFN-γ expression in CD8^+^ T cells in both LNs and tumors was significantly increased, whereas IFN-γ expression was not altered in other immune cells, such as CD4^+^ T cells, macrophages, and DCs after topical IMQ stimulation in the murine tumor model. These results indicate that IFN-γ produced by CD8^+^ T cells may play a crucial role in the antitumor effect induced by topical IMQ in our murine tumor model.

The PD-1 pathway is crucial for inducing T-cell exhaustion, and the expression of PD-1 and its ligands, PD-L1 and PD-L2, has been shown to predict better outcomes by anti-PD-1 inhibitors [51,52,53]. Various kinds of inflammatory and oncogenic signaling have been shown to upregulate the expression of PD-L1 and PD-L2 [54,55,56]. Meier et al. also demonstrated that HIV-derived TLR 7/8 ligands can upregulate PD-L1 expression in human DCs and monocytes [57]. In our study, IMQ stimulation significantly upregulated the expression of not only PD-L1 but also of PD-L2 in myeloid cells in murine spleens as well as in human PBMCs. This upregulated PD-L1/PD-L2 expression in myeloid cells in murine spleens was observed at a relatively shorter time (6 h) after IMQ stimulation, indicating that signaling downstream of TLR 7 directly upregulates PD-L1/PD-L2 expression. NFκB is a major downstream molecule of TLR 7, and we showed that inhibition of NFκB significantly suppressed IMQ-induced PD-L1/PD-L2 expression. Conversely, previous reports demonstrated that NFκB activation upregulates PD-L1/PD-L2 expression [56,58,59]. Collectively, the TLR 7–NFκB signaling pathway may play vital roles in IMQ-induced PD-L1/PD-L2 upregulation, although IMQ-induced cytokine production might also be involved in PD-L1/PD-L2 upregulation. Consistent with the in vitro results, we also discovered that PD-L1/PD-L2 expression in myeloid cells in both LNs and tumors was upregulated by IMQ application in the murine tumor model. Although IFN-γ is the most prominent factor to upregulate PD-L1 expression [38], PD-L1 expression in myeloid cells after stimulation with IMQ in both in vitro and in vivo studies was comparable between the wildtype mice and the IFN-γ-deficient mice. Therefore, our study has revealed that the IMQ-induced antitumor effect is dependent on the increased production of IFN-γ and that IFN-γ is not involved in the IMQ-induced upregulation of PD-L1 expression in myeloid cells.

PD-1 expression in T cells is known to be upregulated following TCR-mediated activation [60,61]. A previous study demonstrated that the common γ-chain cytokines including IL-2 and IL-15, which could be produced by myeloid cells and B cells, also upregulate PD-1 expression in T cells [60]. In our study, PD-1 expression in T cells of splenocytes was not increased after 6 h but increased 24 h after IMQ stimulation in vitro. We confirmed that mRNA expressions including those of Il2 and Il15 were increased 6 h after IMQ stimulation and that protein concentration of IL-2 in the culture supernatants of splenocytes was also increased 24 h after IMQ stimulation. Moreover, the IMQ-induced upregulation of PD-1 in T-cells was abolished by the JAK inhibitor. Given that the common γ-chain cytokine receptors are associated with JAK, the increased cytokine production including common γ-chain cytokines by IMQ stimulation may significantly contribute to the IMQ-induced upregulation of PD-1 expression in T cells. Consistently, we also found upregulation of PD-1 expression in T cells by IMQ application in our murine tumor model.

In our study, combination therapy of topical IMQ and anti-PD-1 antibody showed a potent antitumor effect. This effect might be simply due to the sum of activated innate and adaptive immunity against tumors induced by topical IMQ and anti-PD-1 antibody rather than by a synergistic effect of the combination therapy. However, given that IMQ significantly upregulates PD-1/PD-L1/ PD-L2 expression, which may dampen the IMQ-induced antitumor effect, blockade of IMQ-driven upregulated PD-1 inhibitory signaling by use of anti-PD-1 antibodies may have significantly contributed to the potent antitumor effect of the combination therapy in our study. Thus, this combination therapy could be a reasonable strategy for enhancing the antitumor effect, although there was a lack of direct evidence showing that IMQ-upregulated expression of PD-1/PD-L1/PD-L2 is involved in the antitumor effect of the combination therapy in our study. Irrespective of whether an additive or synergistic effect works in the combination therapy, the potent antitumor effect in our study suggests that combination therapy of topical IMQ plus anti-PD-1 antibody, both of which are already used in clinical settings, is a promising treatment for unresectable cutaneous lesions of various malignancies including malignant melanoma.

Our study was limited by several factors. Since the molecular and cellular phenotypes including immunogenicity in mouse syngeneic tumor models differ from typical human tumors derived from the same tissue of origin, cancer cell lines do not always recapitulate human tumor cells as described above [27,45]. From our study, the antitumor effect of topical IMQ plus anti-PD-1 antibody would be dependent on the immunogenicity of each tumor. However, not only does immunogenicity differ between each tumor, but also it may differ by the clinicopathological type, as reported in melanomas [47,48]. In addition, penetration of IMQ may significantly affect its antitumor effect. Although it has been reported that applied IMQ can penetrate through the murine skin, little is known about the depth and amount of IMQ penetrate the human skin [62]. As we are now planning a clinical trial of this combination therapy, we should carefully consider not only which tumor and type should be included, but also how we deliver IMQ.

## Data Availability

The datasets used and/or analyzed during the current study are available from the corresponding author on reasonable request.

## Supplementary Materials

The following are available online at https://www.mdpi.com/article/10.3390/cancers13163948/s1, Figure S1: Gating strategy for myeloid cells, Figure S2: TLR7 expression in myeloid cells, Figure S3: TRAIL expression in myeloid cells after IMQ stimulation in vitro, Figure S4: PD-L1 and PD-L2 expression in MC38 cells after IMQ stimulation in vitro, Figure S5: *Ccl2* expression of MC38 after IMQ stimulation in vitro, Figure S6: PD-L1 and PD-L2 expression in MC38 cells after IMQ stimulation in vivo, Figure S7: Poor antitumor effect of topical IMQ monotherapy, anti-PD-1 antibody therapy monotherapy, and the combination therapy against B16F10 melanoma.

## Abbreviations

TLR	Toll-like receptor
IMQ	Imiquimod
IFN	Interferon
TRAIL	Tumor necrosis factor-related apoptosis-inducing ligand
ITM	In-transit metastasis
LN	Lymph node
ORR	Overall response rate
BSA	Bovine serum albumin
qRT-PCR	Quantitative reverse transcription-polymerase chain reaction
PBMC	Peripheral blood mononuclear cell
GAPDH	Glyceraldehyde-3-phosphate dehydrogenase
DC	Dendritic cell
NF-κB	Nuclear factor-kappa B
