# Supplementary material for: Combination Treatment of Topical Imiquimod Plus Anti-PD-1 Antibody Exerts Significantly Potent Antitumor Effect
Source: Cancers (Basel). 2021 Aug 5;13(16):3948. doi: 10.3390/cancers13163948 (PMC8391905; doi:10.3390/cancers13163948)
Supplement: Supplementary file 1 [file cancers-13-03948-s001.zip › cancers-1325068-supplementary.pdf]

cancers

MDPI

Combination Treatment of Topical Imiquimod Plus Anti-PD-1 Antibody Exerts Significantly Potent Antitumor Effect

Kazumasa Oya, Yoshiyuki Nakamura, Zhu Zhenjie, Ryota Tanaka, Naoko Okiyama, Yuki Ichimura, Yosuke Ishitsuka, Akimasa Saito, Noriko Kubota, Rei Watanabe, Hideaki Tahara, Manabu Fujimoto and Yasuhiro Fujisawa

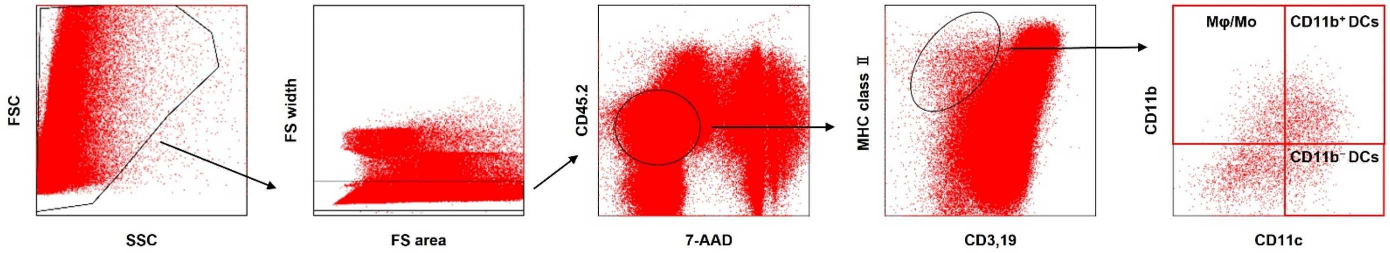

Figure S1. Gating strategy for myeloid cells. Representative flow cytometric gating of murine splenocytes or human peripheral blood mononuclear cells. In brief, single cells were gated according to forward and side scatter, and doublets were excluded. Dead cells were excluded and then CD45.2⁺ cells were gated into CD3⁻CD19⁻MHC class II⁺. Subsequently, these cells were classified into 3 subsets on the basis of CD11b and CD11c expression, as MHC class II⁺ CD11b⁺ CD11c⁻ cells (macrophages [Mφ] in tissues and monocytes in human blood), MHC class II⁺ CD11b⁺ CD11c⁺ cells (CD11b⁺ dendritic cells [DCs]), and MHC class II⁺ CD11b⁻ CD11c⁺ cells (CD11b⁻ DCs). FSC: forward scatter, SSC: side scatter, FS: forward scatter, Mφ: macrophages, Mo: monocytes, DCs: dendritic cells.

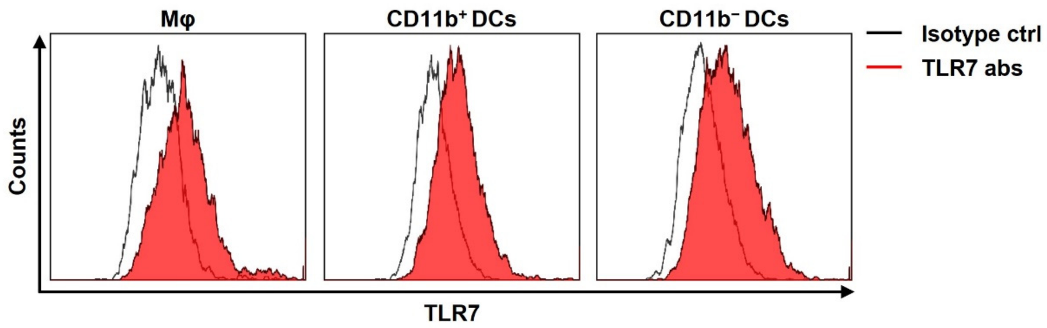

Figure S2. TLR7 expression in myeloid cells. Representative flow cytometric analysis of TLR7 expression in macrophages, CD11b⁺ DCs, and CD11b⁻ DCs of murine spleen. Mφ: macrophages, DCs: dendritic cells, ctrl: control, abs: antibodies.

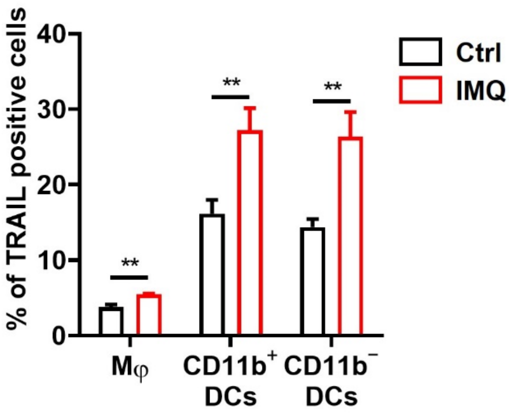

Figure S3. TRAIL expression in myeloid cells after IMQ stimulation *in vitro*. Flow cytometric analysis of TRAIL expression in myeloid cells of murine spleen stimulated with IMQ for 24 hours (*n* =

6 in each group). Mφ: macrophages, DCs: dendritic cells, ctrl: control, Ctrl: control, IMQ: imiquimod, TRAIL: tumor necrosis factor-related apoptosis-inducing ligand. Error bars indicate ±1 SEM; ***p* < 0.01.

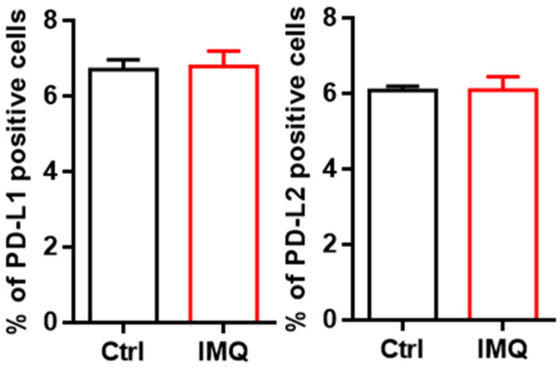

Figure S4. PD-L1 and PD-L2 expression in MC38 cells after IMQ stimulation *in vitro*. Flow cytometric analysis of PD-L1 and PD-L2 expression in MC38 colon cancer cells stimulated with IMQ for 24 hours ($n = 3$ in each group). Ctrl: control, IMQ: imiquimod.

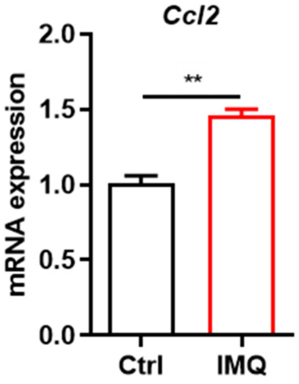

Figure S5. *Ccl2* expression of MC38 after IMQ stimulation *in vitro*. Quantitative RT-PCR analysis of *Ccl2* expression of MC38 colon cancer cells stimulated with IMQ for 24 hours ($n = 6$ in each group). Ctrl: control, IMQ: imiquimod. Error bars indicate ±1 SEM; ** $p < 0.01$.

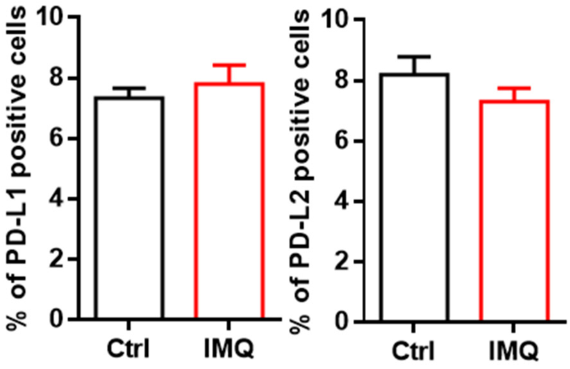

Figure S6. PD-L1 and PD-L2 expression in MC38 cells after IMQ stimulation *in vivo*. Flow cytometric analysis of PD-L1 and PD-L2 expression in MC38 colon cancer cells 5 days after tumor inoculation with IMQ application at day 4 ($n = 6$ in the group of PD-L1 expression

analysis, *n* = 5 in the group of PD-L2 expression analysis). Ctrl: control, IMQ: imiquimod.

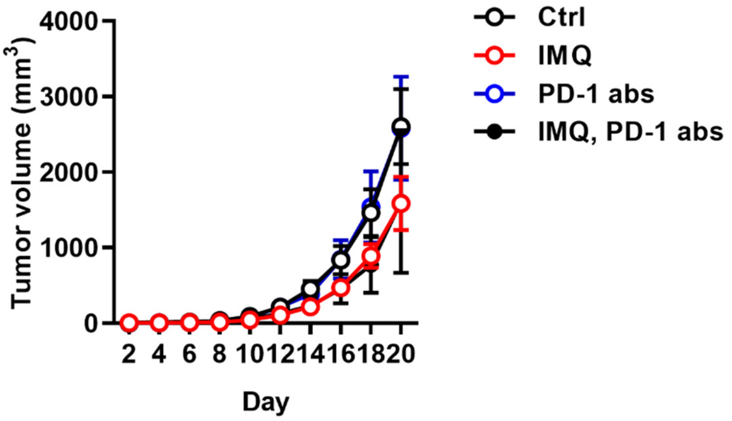

Figure S7. Poor antitumor effect of topical IMQ monotherapy, anti-PD-1 antibody therapy monotherapy, and the combination therapy against B16F10 melanoma. Time course of the tumor volume after 3 × 10^5 B16F10 melanoma cells were intradermally inoculated into the backs of wildtype mice with topical IMQ application, anti-PD-1 antibody treatment, topical IMQ plus anti-PD-1 antibody treatment, or no treatment (*n* = 11 in Ctrl, 12 in IMQ, 6 in PD-1 abs and IMQ, PD-1 abs group). IMQ: imiquimod, Ctrl: control, abs: antibodies. Error bars indicate ±1 SEM.
